# Impact of media brand on cefiderocol disk diffusion results

**DOI:** 10.1128/jcm.01648-24

**Published:** 2025-03-25

**Authors:** M. G. DeMarco, A. M. Field, L. E. Donohue, H. L. Cox, T. S. Kidd, K. E. Barry, A. J. Mathers

**Affiliations:** 1 Department of Pharmacy Services, University of Virginia Health 12350 , Charlottesville, Virginia, USA; 2 Division of Infectious Diseases and International Health, School of Medicine, University of Virginia 12349, Charlottesville, Virginia, USA; 3 Clinical Microbiology, University of Virginia Health 12350 , Charlottesville, Virginia, USA; Johns Hopkins University, Baltimore, Maryland, USA

**Keywords:** cefiderocol, disk diffusion, media, susceptibility testing, quality control, antibiotic resistance, *Pseudomonas aeruginosa*, *Acinetobacter baumannii*, *Stenotrophomonas*, Carbapenem-resistant Enterobacterales

## Abstract

Cefiderocol is a siderophore cephalosporin that utilizes iron transport systems to cross cell membranes. This unique strategy complicates antimicrobial susceptibility testing (AST) due to variable iron content in media. While guidance for using iron-depleted media exists for broth microdilution (BMD), disk diffusion (DD) with commercial media is a common AST method in the clinical laboratory. We investigated cefiderocol DD result variability using multiple Mueller–Hinton agar (MHA) brands. DD results using Remel (Thermo Fisher Scientific, San Diego, CA), Hardy (Hardy Diagnostics, Springboro, OH), and BBL (Becton Dickinson, East Rutherford, NJ) MHA were compared to those of BMD using iron-depleted cation-adjusted Mueller–Hinton broth. BMD reproducibility, BMD trailing endpoints, and DD intra- and inter-brand variability in zones of inhibition were investigated. Forty-seven multidrug-resistant clinical isolates and three Antibiotic Resistance Bank isolates composed of *Pseudomonas aeruginosa*, carbapenemase (CP–) and non-carbapenemase-producing (non-CP–) carbapenem-resistant Enterobacterales (CRE), *Acinetobacter baumannii* complex, *Stenotrophomonas maltophilia*, and *Burkholderia cepacia* complex were tested. Categorical agreement (CA) ≥ 90% was only demonstrated using CLSI breakpoints with BBL agar. Intra- and inter-brand variability in DD were highest for *P. aeruginosa* and CRE, with 25% (5/20) and 16.7% (3/18) exhibiting discrepant AST interpretations, respectively. Isolates not susceptible to cefiderocol via BMD were commonly associated with AST interpretation errors and lower CA. Using commercial MHA for DD resulted in frequent AST interpretation discrepancies, particularly for isolates that were not susceptible to cefiderocol by BMD. Methods and quality control may need to be revisited to ensure the reliability of DD for cefiderocol AST.

**IMPORTANCE:**

The novel mechanism of action of cefiderocol overcomes a variety of resistance mechanisms associated with gram-negative bacteria and positions the agent as an attractive option for treating infections involving multidrug-resistant pathogens. The availability of accurate, timely antimicrobial susceptibility testing methods for cefiderocol in clinical microbiology laboratories is critical as cefiderocol-resistant isolates have been described and may contribute to treatment failure. Iron-depleted broth microdilution testing may not be feasible for use in many clinical laboratories. While disk diffusion is an appealing, practical method to implement, our data demonstrate reproducibility issues across agar brands, most notably for organisms that do not test susceptible to cefiderocol when using broth microdilution. Discrepancy errors and misclassifications of resistant isolates as susceptible, and susceptible isolates as resistant, may mislead clinicians and compromise the treatment efficacy. More work is needed to standardize practical yet reproducible methods for cefiderocol antimicrobial susceptibility testing.

## INTRODUCTION

Cefiderocol is a promising therapeutic agent for difficult-to-treat and multidrug-resistant (MDR) gram-negative (GN) bacterial infections due to its novel structure composed of a cephalosporin moiety joined to a siderophore. This siderophore provides a unique cell entry method that utilizes bacterial iron transport systems to circumvent the low outer membrane permeability. It is currently approved by the U.S. Food and Drug Administration (FDA) for use in patients 18 years of age or older for the treatment of complicated urinary tract infections and hospital-acquired or ventilator-associated pneumonia caused by susceptible GN pathogens (1).

While the mechanism of action of cefiderocol helps overcome some antimicrobial resistance and takes advantage of the low iron environment of infected tissues, demonstrating reproducible antimicrobial susceptibility testing (AST) *in vitro* creates challenges for both broth microdilution (BMD) and disk diffusion (DD) modalities (2). Concerns related to the reproducibility of cefiderocol AST between BMD and DD have been reported (3) and may relate to a lack of standardized iron content in commercial media (2). Methods to deplete iron from broth used for BMD have been described (4–6) but are imperfect and impractical for application in many clinical laboratories. Analogous guidance does not exist for DD performed with commercially available Mueller–Hinton agar (MHA) plates as the bound state of iron in the media is thought to result in an iron-depleted environment (7, 8). However, recent evidence suggests that results of cefiderocol AST by DD may vary by the medium brand (9). Additionally, differences in cefiderocol clinical breakpoints from the Clinical and Laboratory Standards Institute (CLSI) (4), the European Committee on Antimicrobial Susceptibility Testing (EUCAST) (10), and the FDA (11) have contributed to discordant susceptibility interpretations. Since clinical failure and treatment-emergent resistance have been associated with the use of cefiderocol (12–16), reliable and accurate AST is critical for guiding effective treatment decision-making.

We sought to investigate the impact of the MHA brand on the interpretation of DD results for a collection of MDR GN bacterial isolates across multiple clinical breakpoint standards and to assess the concordance of these interpretations with those of BMD, which served as the reference method.

## MATERIALS AND METHODS

### Bacterial isolates

Fifty unique GN bacterial isolates, including 47 de-identified MDR organisms collected from discarded clinical samples between June 2021 and February 2023 (University of Virginia Medical Center Clinical Microbiology Laboratory, Charlottesville, VA) and three Antibiotic Resistance Bank isolates (Centers for Disease Control and Prevention, Atlanta, GA) were utilized for BMD and DD testing. Isolates were stored at −80°C.

### Broth microdilution

**Fig 1:**
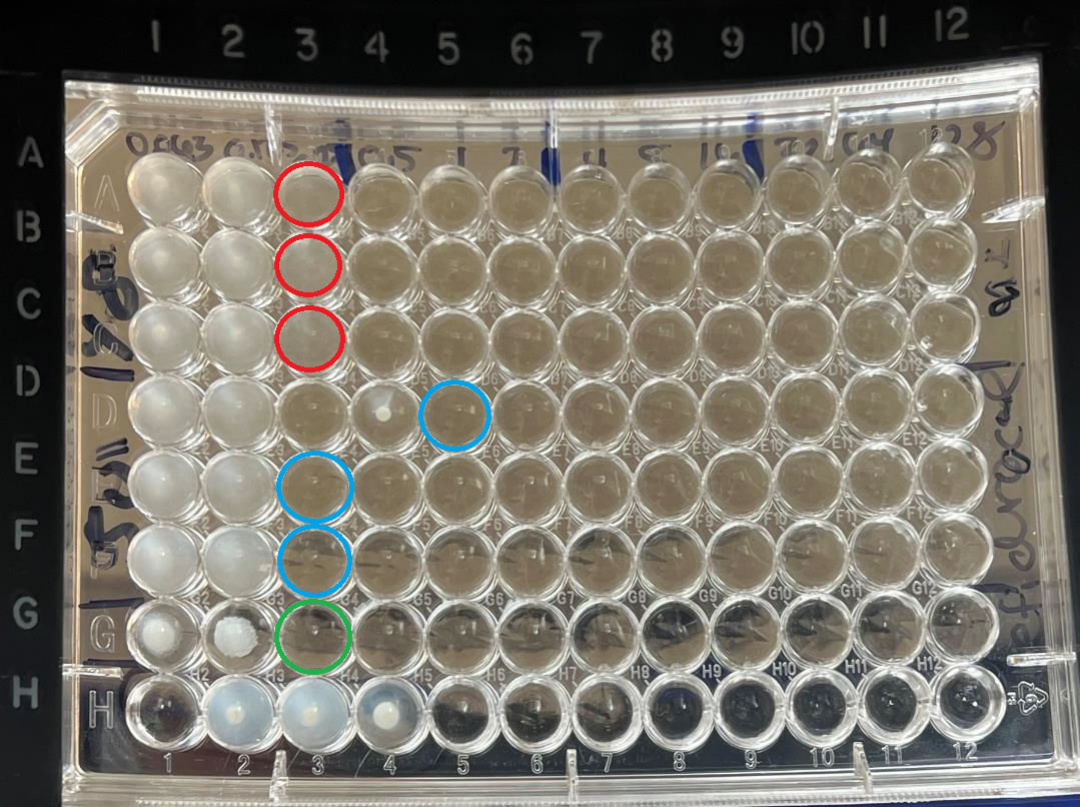
Cefiderocol BMD testing with clear vs trailing MIC endpoints. Rows A–C = *Stenotrophomonas maltophilia* isolate (Sm2) with hazy trailing endpoints. Red circles denote MICs. Rows D–F = *Stenotrophomonas maltophilia* isolate (Sm4) with clear endpoints. Blue circles denote MICs. Row D featured one skipped well. Row G = *E. coli* ATCC 25922 positive control. Green circle denotes the MIC. Row H = Negative control and growth controls for both *Stenotrophomonas maltophilia* isolates and *E. coli* ATCC 25922.

BMD was performed with plates containing cefiderocol (MedChemExpress, Monmouth Junction, NJ) and iron-depleted cation-adjusted Mueller–Hinton broth (BBL, Benton Dickerson and Co, Franklin Lakes, NJ) (ID CAMHB) prepared following CLSI guidelines (4, 5). The final iron concentration of the media (less than 0.03 mg/L) was verified using the Visocolor HE Iron assay (Macherey-Nagel, Duren Germany). Specifically, each isolate was combined with ID CAMHB broth to prepare a consistent inoculum of 0.5 McFarland standard (approximately 10^8^ CFU/mL) via the colony suspension method. Then, each standard was diluted with ID CAMHB to produce a 1:100 dilution to approximate a final concentration of 10^6^ CFU/mL. Fifty microliters from each isolate was combined with 50 µL of cefiderocol solutions ranging from 0.063 µg/mL to 128 µg/mL and repeated across three rows using one inoculum per isolate in an effort to minimize the variability. Each BMD plate also contained a row of *Escherichia coli* ATCC 25922 as a quality control (QC) with in-range minimum inhibitory concentration (MIC), a negative control with ID CAMHB broth only, a growth control (GC) well for each isolate containing no cefiderocol, and a GC well for *E. coli* ATCC 25922 containing no cefiderocol. Plates were incubated at 35 ˚C for 16–20 hours before being read according to CLSI guidelines (4, 5). BMD results were accepted for isolates demonstrating adequate growth in the form of a button of ≥2 mm or heavy turbidity in each GC well and no more than one skipped well per row. MICs for acceptable organisms with clear endpoints were recorded as the the lowest concentration of cefiderocol with no growth observed, as shown in Fig. 1. For organisms with trailing endpoints of faint growth across multiple wells, MICs were recorded as the lowest concentration of cefiderocol where a button of ≤1 mm, a light haze, or faint turbidity was observed, as shown in Fig. 1. Modal MICs for each isolate were calculated and interpreted according to available CLSI, EUCAST, and FDA clinical breakpoints when available for each species (Table 1) (4, 10, 11).

**TABLE 1:** Cefiderocol susceptibility testing interpretive criteria

Organism	CLSI*^a^*			EUCAST*^b^*		FDA*^c^*		
	S	I	R	S	R	S	I	R
Interpretive categories and MIC breakpoints (μg/mL)								
Enterobacterales	≤ 4	8	≥ 16	≤ 2	> 2	≤ 4	8	≥ 16
*Pseudomonas aeruginosa*	≤ 4	8	≥ 16	≤ 2	> 2	≤ 1	2	≥ 4
*Acinetobacter baumannii* complex	≤ 4	8	≥ 16	Insufficient evidence		≤ 1	2	≥ 4
*Stenotrophomonas maltophilia*	≤ 1	No breakpoints available				No breakpoints available		
*Burkholderia cepacia* complex	No breakpoints available							
Interpretive categories and zone diameter breakpoints (mm)								
Enterobacterales	≥ 16	9–15	≤ 8	≥ 23	< 23	≥ 16	9–15	≤ 8
*Pseudomonas aeruginosa*	≥ 18	13–17	≤ 12	≥ 22	< 22	≥ 22	13–21	≤ 12
*Acinetobacter baumannii* complex	≥ 15	Do not report if ≤14		≥ 17	Insufficient evidence	≥ 19	12–18	≤ 11
*Stenotrophomonas maltophilia*	≥ 15	No breakpoints available		≥ 20		No breakpoints available		
*Burkholderia cepacia* complex	No breakpoints available							

^*a*^ Clinical and Laboratory Standards Institute Performance Standards for Antimicrobial Susceptibility Testing, 34th ed.

^*b*^ European Committee on Antimicrobial Susceptibility Testing Breakpoint tables for interpretation of MICs and zone diameters, version 14.0.

^*c*^ United States Food and Drug Administration Identified Breakpoints for Cefiderocol Injection, last updated 31 January 2023.

### Disk diffusion

DD was performed in accordance with CLSI guidelines using 30 µg cefiderocol disks (Hardy Diagnostics, Springboro, OH) and three brands of MHA: Remel (Thermo Fisher Scientific, San Diego, CA), Hardy (Hardy Diagnostics, Springboro, OH), and BBL (Becton Dickinson, East Rutherford, NJ) (4, 17). QC was performed by plating *E. coli* ATCC 25922 and *Pseudomonas aeruginosa* ATCC 27853 on Remel, Hardy, and BBL MHA. Each isolate was incubated at 37°C on three MHA plates from each brand (for a total of nine plates per isolate) using 0.5 McFarland standard preparations (17). *Stenotrophomonas maltophilia* isolates were incubated for 20–24 hours, and all others were incubated for 16–18 hours. Mean zones of inhibition for each isolate per plate brand were calculated and interpreted using available CLSI, EUCAST, and FDA clinical breakpoints by species (Table 1) (4, 10, 11). If inner colonies were observed within the zone of inhibition, the size of the colony-free inner zone was measured and reported. Per organism, intra-brand variability was calculated by comparing the mean zones of inhibition between the three plates repeated for each brand. Inter-brand variability in terms of range deviation was calculated by comparing the mean zones of inhibition across MHA plate brands.

### Agreement analysis

Categorical agreement (CA), minor errors (mE), major errors (ME), and very major errors (VME) were assessed by comparing CLSI clinical breakpoint interpretations for mean zones of inhibition determined via DD per brand of MHA to modal MICs determined via BMD as the reference method (4). Acceptable thresholds for agreement were defined following CLSI guidelines, with CA ≥ 90%, mE ≤10%, and ME and VME < 3% (18–20). Targets for acceptable discrepancy rate criteria via the error rate-bounded method were also used in accordance with CLSI guidelines (19, 20).

## RESULTS

### Bacterial isolates

**Fig 2:**
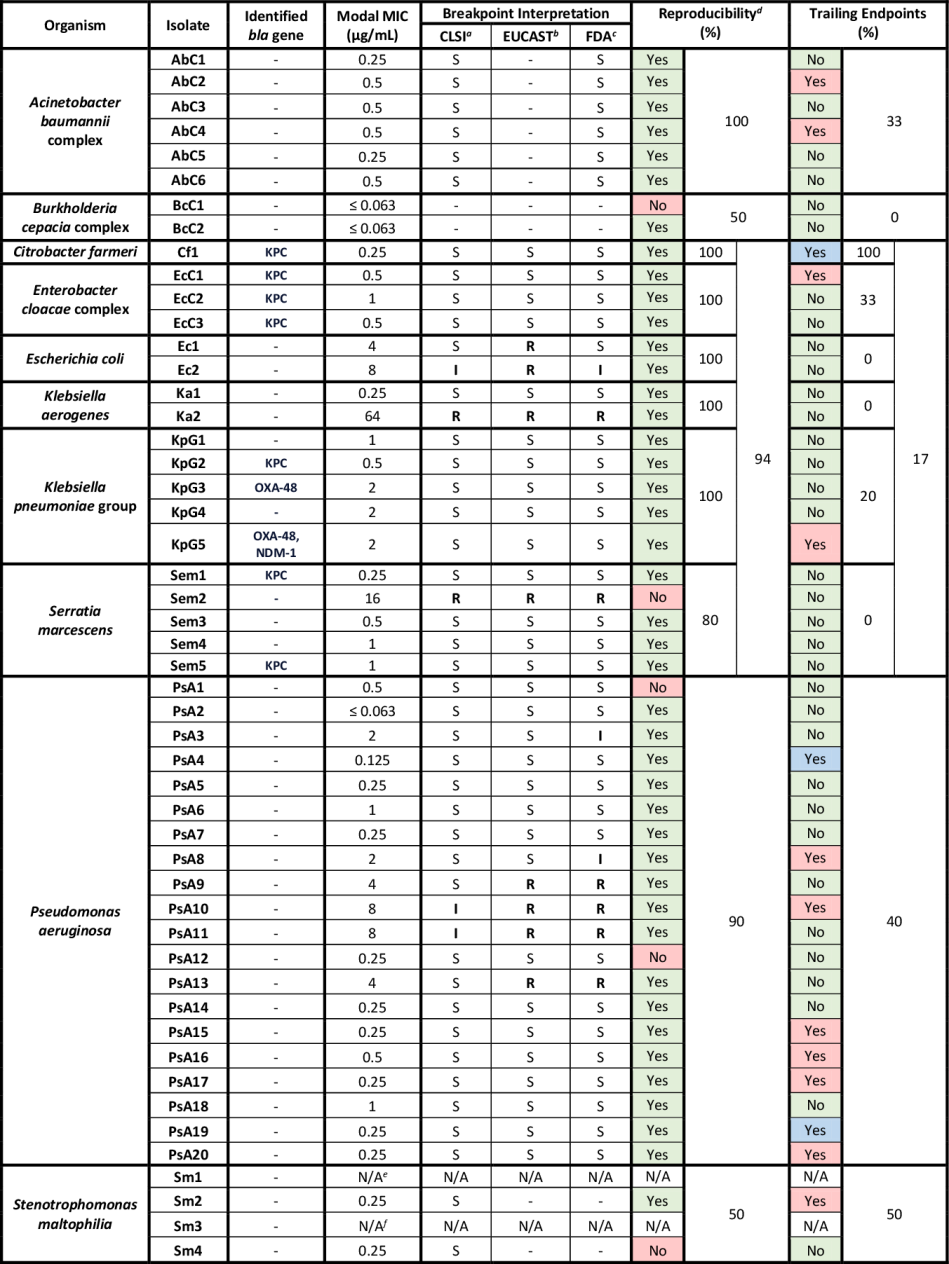
Cefiderocol BMD results. AbC, *Acinetobacter baumannii* complex; BcC, *Burkholderia cepacia* complex; Cf, *Citrobacter farmeri*; EcC, *Enterobacter cloacae* complex; Ec, *Escherichia coli*; I, intermediate; Ka, *Klebsiella aerogenes*; KPC, *Klebsiella pneumoniae* carbapenemase; KpG, *Klebsiella pneumoniae* group; MIC, minimum inhibitory concentration; NDM, New Delhi metallo-beta-lactamase; OXA-48, oxacillinase-48 carbapenemase; PsA, *Pseudomonas aeruginosa*; R, resistant; Sem, *Serratia marcescens*; Sm, *Stenotrophomonas maltophilia*; S, susceptible. Green shading represents acceptable reproducibility and lack of observed trailing endpoints. Red shading represents lack of reproducibility and observation of trailing endpoints. Blue shading represents isolates with both trailing endpoints observed with broth microdilution and inner colonies observed with disk diffusion. *^a^*Clinical and Laboratory Standards Institute Performance Standards for Antimicrobial Susceptibility Testing, 34th ed. *^b^*European Committee on Antimicrobial Susceptibility Testing Breakpoint tables for interpretation of MICs and zone diameters, version 14.0. *^c^*United States Food and Drug Administration Identified Breakpoints for Cefiderocol Injection, last updated 31 January 2023. *^d^*Calculated as the number of isolates with all MICs within one doubling dilution of the modal MIC divided by all isolates per organism species. *^e^*Isolate did not demonstrate growth on the BMD test and control wells. *^f^*Isolate was not available for BMD testing.

The 50 unique bacterial isolates tested were composed of clinical MDR *Pseudomonas aeruginosa* (*n* = 20), carbapenemase (CP–) and non-carbapenemase-producing (non-CP–) carbapenem-resistant Enterobacterales (CRE) (*n* = 18, *n* = 9 CP-CRE, *n* = 9 non-CP-CRE), *Acinetobacter baumannii* complex (*n* = 6, *n* = 3 from the Antibiotic Resistance bank), *Stenotrophomonas maltophilia* (*n* = 4), and *Burkholderia cepacia* complex (*n* = 2) (Fig. 2).

### Broth microdilution

BMD testing was successfully performed on 48 isolates (Fig. 2). Of the 46 isolates that underwent BMD testing and for which cefiderocol clinical breakpoints exist (i.e., excluding *B. cepacia* complex), most demonstrated *in vitro* susceptibility, including all *A. baumannii* complex and tested *S. maltophilia* isolates. Intermediate or resistant interpretations per CLSI breakpoints (*n* = 5/46, 10.9%) were recorded for one *Escherichia coli* (Ec2), one *Klebsiella aerogenes* (Ka2), one *Serratia marcescens* (Sem2), and two *P. aeruginosa* (PsA10 and PsA11) isolates. Ten isolates (*n* = 10/46, 22%) demonstrated a modal MIC interpreted as intermediate or resistant by at least one set of established clinical breakpoints (Table 1): two *E. coli* (Ec1 and Ec2), one *K. aerogenes* (Ka2), one *S*. *marcescens* (Sem2), and six *P. aeruginosa* (PsA3, PsA8, PsA9, PsA10, PsA11, and PsA13). No CP-CRE isolates were interpreted as intermediate or resistant. BMD results for most isolates were within one twofold dilution of their respective modal MICs, demonstrating low variability. Trailing endpoints (Fig. 1) were observed for fourteen isolates, including *S. maltophilia* (Sm2; *n* = 1/2; 50%), *P. aeruginosa* (PsA4, PsA8, PsA10, PsA15–17, PsA19, and PsA20; *n* = 8/20; 40%), *A. baumannii* complex (AbC2 and AbC4; *n* = 2/6; 33%), CP-*C. farmeri* (Cf1; *n* = 1/1; 100%), CP-*E. cloacae* complex (EcC1; *n* = 1/3; 33%), and CP-*K. pneumoniae* group (KpG5; *n* = 1/5; 20%).

### Disk diffusion

**Fig 3:**
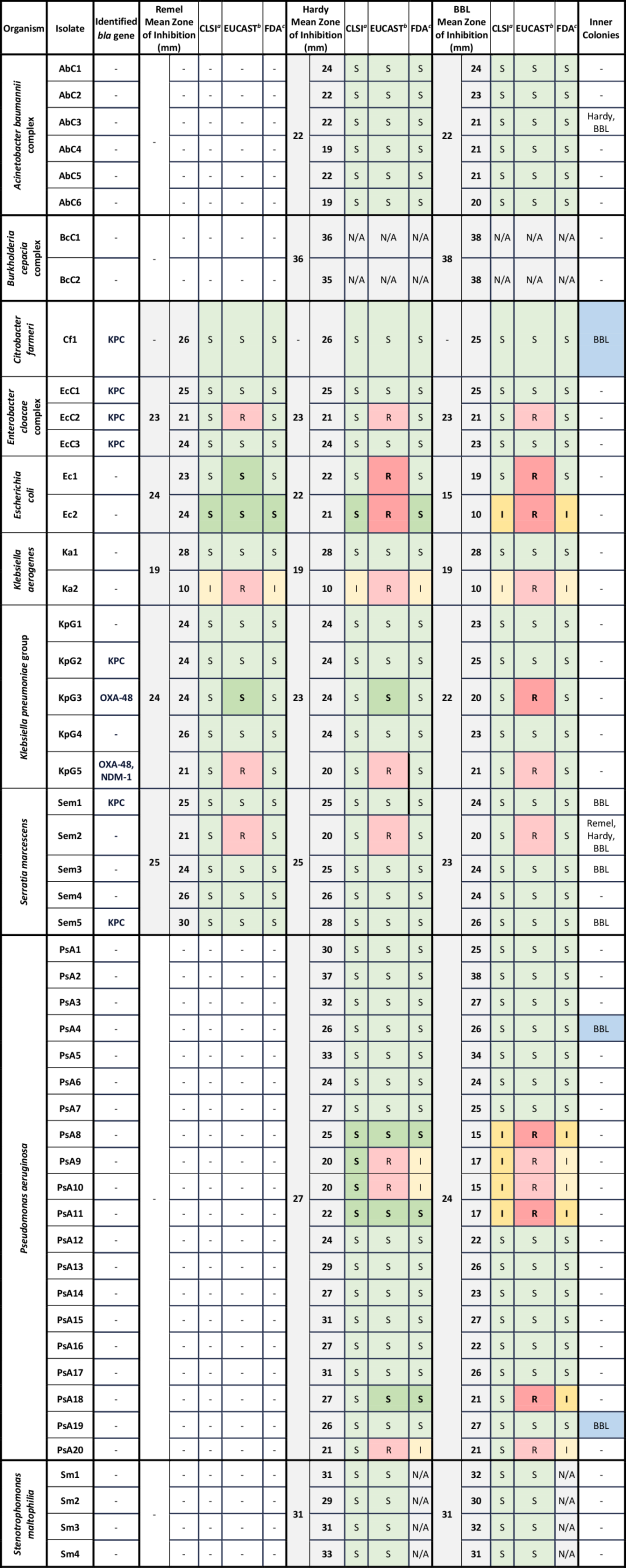
Inter-brand variability in DD mean zones of inhibition with cefiderocol clinical breakpoint interpretations. AbC, *Acinetobacter baumannii* complex; BcC, *Burkholderia cepacia* complex; Cf, *Citrobacter farmeri*; DD, disk diffusion; EcC, *Enterobacter cloacae* complex; Ec, *Escherichia coli*; I, intermediate; Ka, *Klebsiella aerogenes;* KPC, *Klebsiella pneumoniae* carbapenemase; KpG, *Klebsiella pneumoniae* group; NDM, New Delhi metallo-beta-lactamase; OXA-48, oxacillinase-48 carbapenemase; PsA, *Pseudomonas aeruginosa*; R, resistant; Sem, *Serratia marcescens*; Sm, *Stenotrophomonas maltophilia*; S, susceptible *^a^*Clinical and Laboratory Standards Institute Performance Standards for Antimicrobial Susceptibility Testing, 34th ed. *^b^*European Committee on Antimicrobial Susceptibility Testing Breakpoint tables for interpretation of MICs and zone diameters, version 14.0. *^c^*United States Food and Drug Administration Identified Breakpoints for Cefiderocol Injection, last updated 31 January 2023.

**Fig 4:**
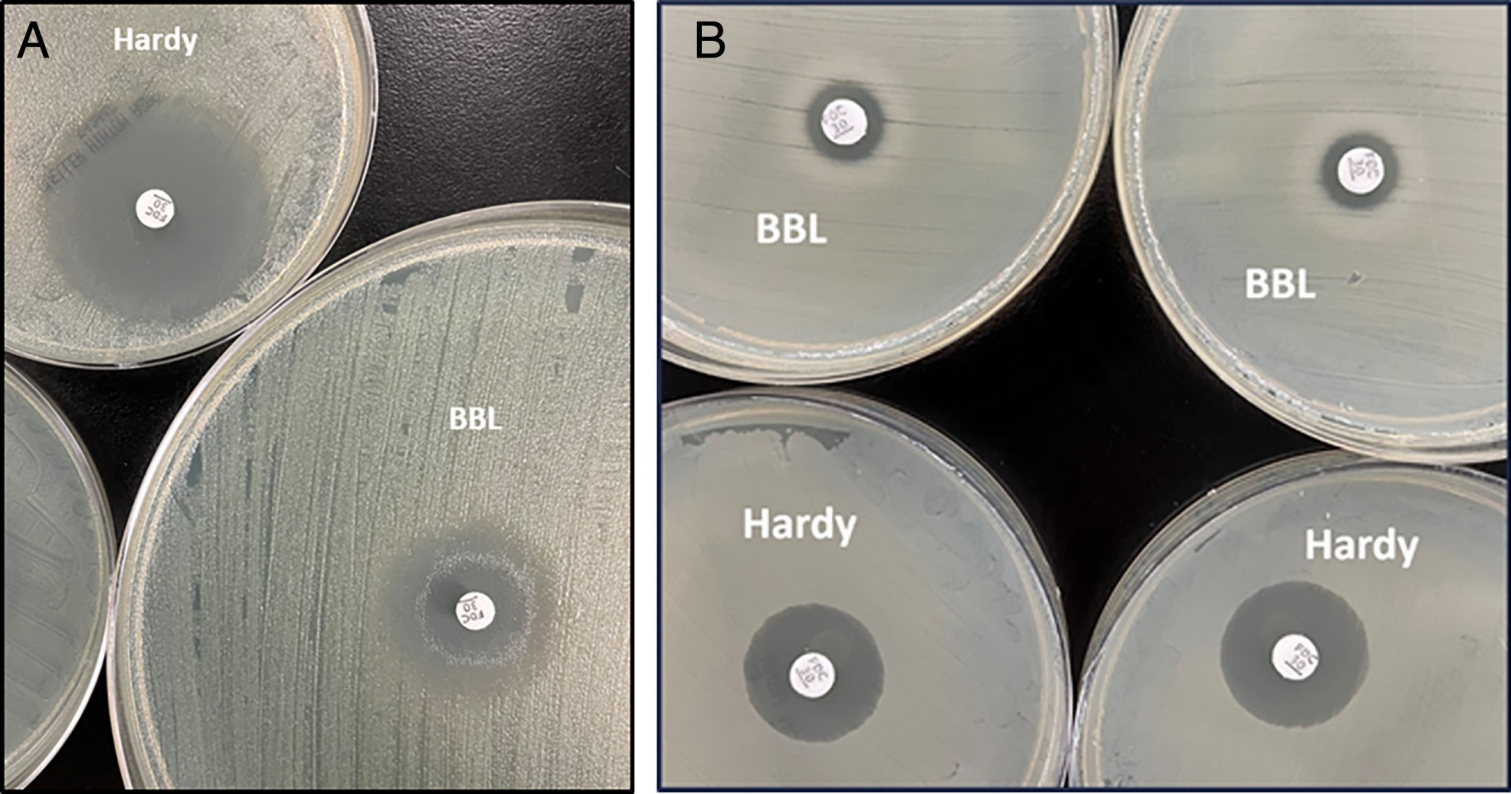
(A). Inter-brand variability in DD zones of inhibition for a *Pseudomonas aeruginosa* isolate (PsA8). Left: Hardy MHA. Right: BBL MHA. (B). Inter-brand variability in DD zones of inhibition for a carbapenem-resistant *E. coli* (no carbapenemase detected) isolate (Ec2). Top row: BBL MHA. Bottom row: Hardy MHA.

Zones of inhibition and interpretive categorization are outlined in Fig. 3. Distributions of zones of inhibition for each species and MHA brand are shown in Table S1-S11. Zones of inhibition for non-Enterobacterales isolates are not reported for Remel MHA as the QC DD test using *P. aeruginosa* ATCC 27853 on Remel MHA failed with a zone of inhibition larger than the reference range. Inner colonies were observed for eight isolates, namely, *A. baumannii* complex (AbC3), *C. farmeri* (Cf1), *S. marcescens* (Sem1, Sem2, Sem3, and Sem5), and *P. aeruginosa* (PsA4 and PsA19). Aside from two isolates (AbC3 and Sem2) for which inner colonies were observed across tested media brands, this phenomenon was limited to BBL MHA. Three isolates demonstrated both DD inner colonies and BMD trailing endpoints (Cf1, PsA4, and Ps19, 3/8, 38%), but were not associated with inter-brand DD interpretation discrepancies. However, of the fourteen isolates with trailing endpoints via BMD, only two were associated with MHA brand variability during DD testing (PsA8 and PsA10, 2/14, 14%). The average intra-brand variability across replicates was minimal for all sub-groups tested: *S. maltophilia* (0.2 mm), *A. baumannii* complex (0.4 mm), CRE (0.6 mm), *P. aeruginosa* (1.1 mm), and *B. cepacia* complex (1.3 mm). The average inter-brand variability across media brands was typically greater: *S. maltophilia* (0.6 mm), *A. baumannii* complex (0.8 mm), CRE (0.9 mm), *P. aeruginosa* (1.9 mm), and *B. cepacia* complex (1.3 mm). Species with higher proportions of isolates that were not susceptible to cefiderocol via BMD, such as *E. coli* (Fig. 2), were more likely to demonstrate larger differences in mean zones of inhibition via DD observed across MHA brands (Remel 24 mm, Hardy 22 mm, and BBL 15 mm) compared to species with a collection of isolates that were more commonly susceptible to cefiderocol via BMD, such as *A. baumannii* complex (Hardy 22 mm and BBL 22 mm), as shown in Fig. 3. For individual isolates, Ec2 demonstrated the largest absolute range (14 mm between Remel and BBL), followed by PsA8 (10 mm between Hardy and BBL). Figure 4A and B demonstrate noticeable inter-brand variability on examples of *P. aeruginosa* and *E. coli* isolates, respectively.

Of the 41 isolates that were categorized as susceptible to cefiderocol by BMD via CLSI breakpoints, eight (19.5%) were not susceptible to cefiderocol by DD across MHA brands when applying CLSI, FDA, and/or EUCAST breakpoints. These discordant isolates included one CP-*E. cloacae* complex (EcC2; *n* = 1/3; 33%), one non-CP-*E. coli* (Ec1; *n* = 1/2; 50%), two CP-*K. pneumoniae* group (KpG3 and KpG5; *n* = 2/5; 40%), and four *P. aeruginosa* (PsA8, PsA9, PsA18, and PsA20; *n* = 4/20; 20%) mostly using EUCAST and FDA breakpoints. Of the *P. aeruginosa* isolates that were susceptible to cefiderocol via BMD with CLSI breakpoints, 2/18 (11%) were not susceptible to cefiderocol by DD testing when applying BBL MHA and CLSI breakpoints compared to 0/18 when using Hardy MHA. All fifteen CRE isolates interpreted as susceptible to cefiderocol per CLSI MIC breakpoints (including all CP-CRE) were susceptible per CLSI disk correlates across MHA brands.

Of the five isolates that were categorized as non-susceptible to cefiderocol by BMD when applying CLSI breakpoints, four (80%) tested susceptible to cefiderocol by DD across MHA brands and interpretation standards. These discordant isolates included one non-CP-*E. coli* isolate (Ec2) that tested susceptible when applying DD using Remel and Hardy MHA; one non-CP-*S. marcescens* isolate (Sem2) that tested susceptible using Remel, Hardy, and BBL MHA; and two *P. aeruginosa* isolates (PsA10 and PsA11) that tested susceptible using Hardy MHA. The only isolate with concordant and not susceptible interpretations by both MIC and DD breakpoints was a non-CP *K. aerogenes* (Ka2), adjudicated as resistant per CLSI MIC and EUCAST zone diameter breakpoints, but intermediate per CLSI and FDA disk correlates. Notably, all *A. baumannii* complex and *S. maltophilia* isolates demonstrated susceptibility to cefiderocol by DD across tested MHA brands and interpretive criteria, consistent with available BMD results.

### Agreement analysis

**Fig 5:**
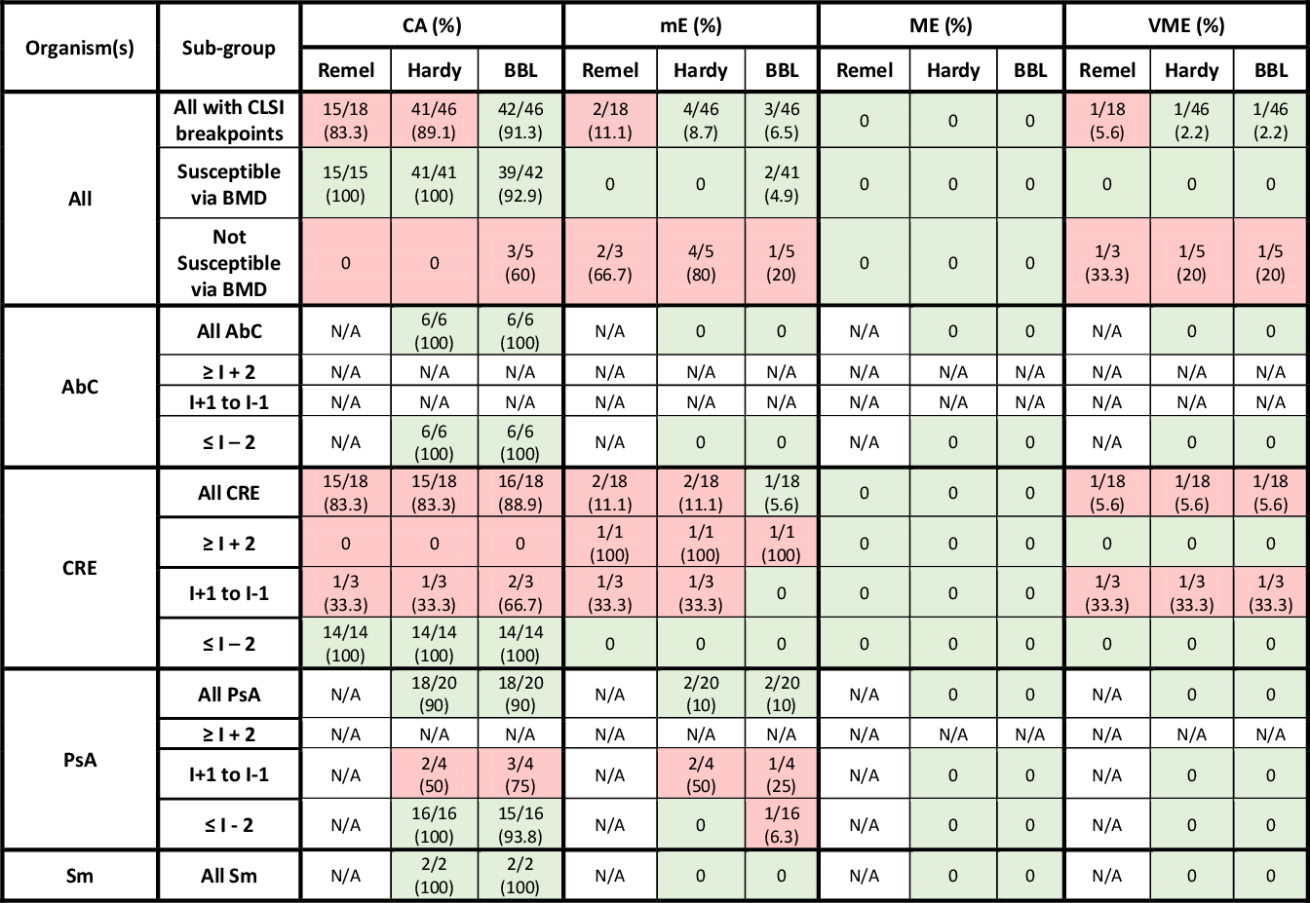
Performance of Remel, Hardy, and BBL MHA DD vs BMD for organisms with CLSI breakpoints*^a^*. AbC, *Acinetobacter baumannii* complex; BMD, broth microdilution; CA, categorical agreement; CRE, carbapenem-resistant *Enterobacterales*; DD, disk diffusion; I, intermediate; mE, minor error; ME, major error; MHA, Mueller–Hinton agar; PsA, *Pseudomonas aeruginosa*; R, resistant; Sm, *Stenotrophomonas maltophilia*; VME, very major error. Green shading represents acceptable agreement or error based on CLSI M23 and M52. Red shading represents unacceptable agreement or error based on CLSI M23 and M52. *^a^*Clinical and Laboratory Standards Institute Performance Standards for Antimicrobial Susceptibility Testing, 34th ed.

**Fig 6:**
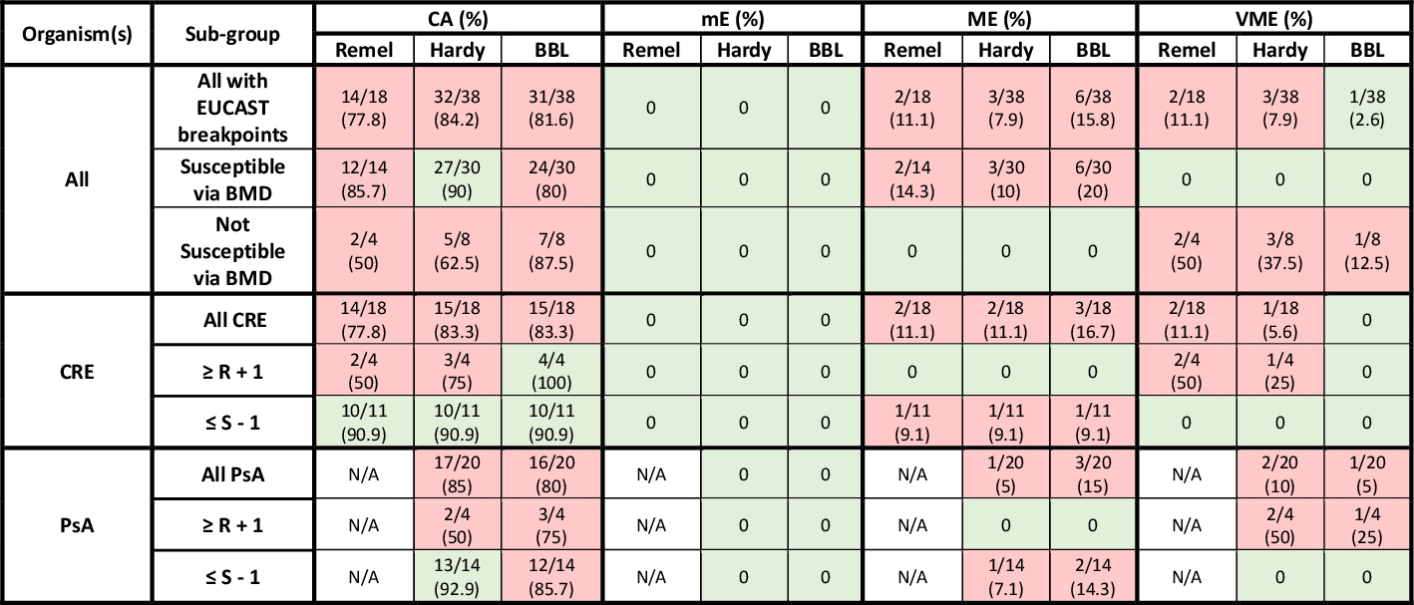
Performance of Remel, Hardy, and BBL MHA DD vs BMD for organisms with EUCAST breakpoints*^a^*. BMD, broth microdilution; CA, categorical agreement; CRE, carbapenem-resistant *Enterobacterales*; DD, disk diffusion; mE, minor error; ME, major error; MHA, Mueller–Hinton agar; PsA, *Pseudomonas aeruginosa*; R, resistant; VME, very major error. Green shading represents acceptable agreement or error based on CLSI M23 and M52. Red shading represents unacceptable agreement or error based on CLSI M23 and M52. *^a^*European Committee on Antimicrobial Susceptibility Testing Breakpoint tables for interpretation of MICs and zone diameters, version 14.0.

**Fig 7:**
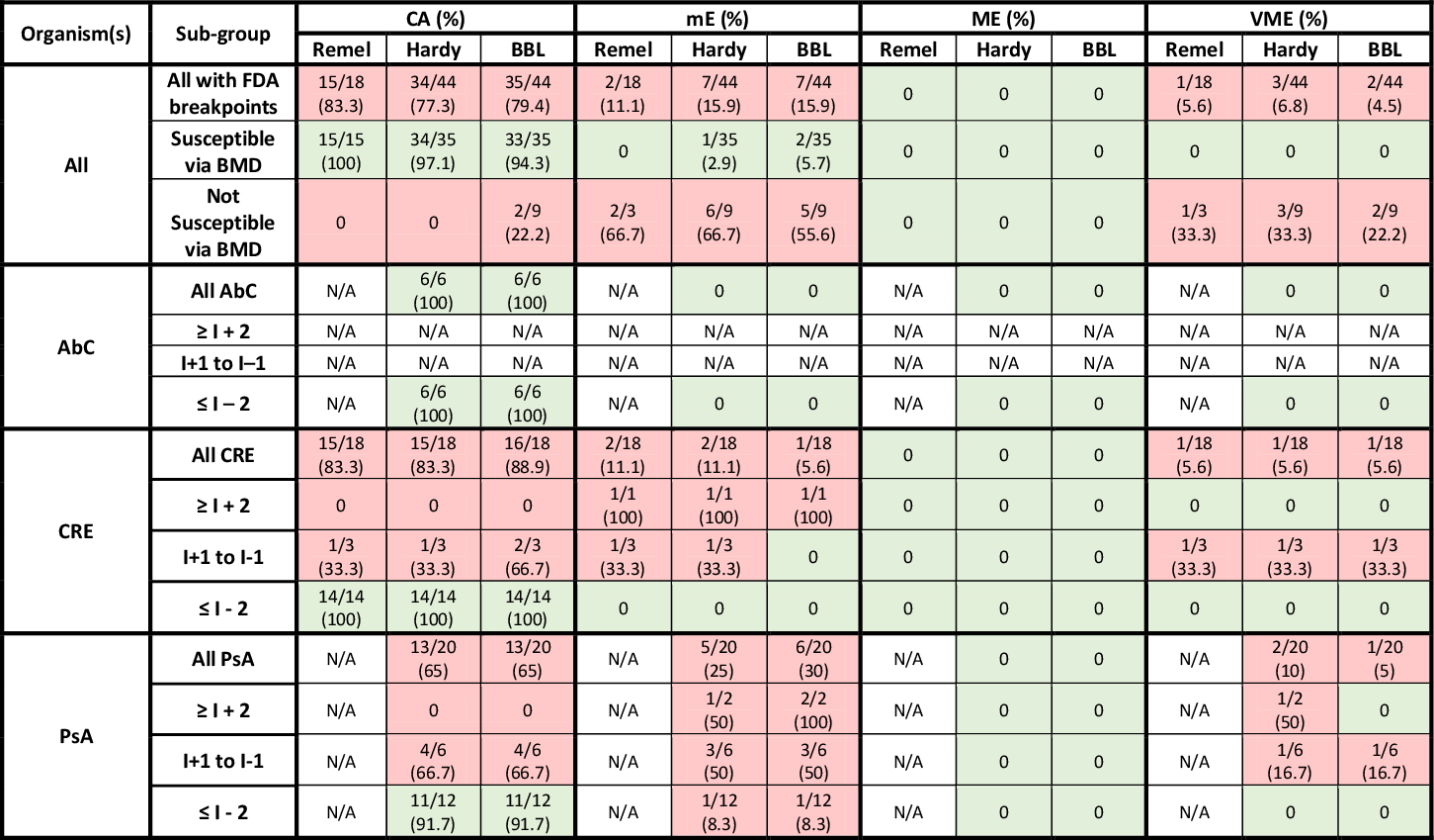
Performance of Remel, Hardy, and BBL MHA DD vs BMD for organisms with FDA breakpoints*^a^*. AbC, *Acinetobacter baumannii* complex; BMD, broth microdilution; CA, categorical agreement; CRE, carbapenem-resistant *Enterobacterales*; DD, disk diffusion; I, intermediate; mE, minor error; ME, major error; MHA, Mueller–Hinton agar; PsA, *Pseudomonas aeruginosa*; R, resistant; VME, very major error. Green shading represents acceptable agreement or error based on CLSI M23 and M52. Red shading represents unacceptable agreement or error based on CLSI M23 and M52. *^a^*United States Food and Drug Administration Identified Breakpoints for Cefiderocol Injection, last updated 31 January 2023.

Performance characteristics of DD interpretations compared to BMD as the reference varied across MHA brands when using CLSI, EUCAST, and FDA clinical breakpoints for cefiderocol (Table 1), as shown in Fig. 5 to 7, respectively.

### CLSI breakpoints

Among isolates that were susceptible to cefiderocol by BMD, CA exceeded 90% for all MHA brands (Fig. 5): Remel (15/15), Hardy (41/41), and BBL (39/41, 95%), with two mEs among *P. aeruginosa* isolates (PsA8 and PsA9). However, CA decreased below 90% among five isolates that were not susceptible to cefiderocol via BMD when compared to DD using any MHA brand: Remel (0/3), Hardy (0/5), and BBL (3/5, 60%). Among our collection of CRE isolates, ≥90% CA was not achieved regardless of the BMD AST interpretation (Remel 15/18, 83%; Hardy 15/18, 83%; BBL 16/18, 89%). However, CA was observed in ≥90% of *P. aeruginosa* isolates (Hardy and BBL both 18/20, 90%).

### EUCAST breakpoints

Thirty isolates were considered susceptible to cefiderocol by BMD using EUCAST breakpoints (Fig. 6). CA was observed in ≥90% of these isolates when using Hardy (27/30, 90%) MHA, but not Remel (12/14, 86%) or BBL (24/30, 80%). No mEs or VMEs were observed, but MEs were common. The isolates that were not susceptible by BMD fared worse, with CA observed in only 2/4 (50%) isolates with Remel MHA, 5/8 (63%) with Hardy MHA, and 7/8 (88%) with BBL MHA. This was driven by VMEs across all MHA brands. Considering errors among all CRE isolates, CA did not exceed 90% when using Remel (14/18, 78%), Hardy (15/18, 83%), or BBL MHA (15/18, 83%) due to MEs and VMEs. Among all *P. aeruginosa* isolates, CA decreased below 90% when using Hardy (17/20, 85%) and BBL (16/20, 80%) MHA due to MEs and VMEs.

### FDA breakpoints

Among 35 isolates that were susceptible to cefiderocol by BMD using FDA interpretations, CA exceeded 90% across all MHA brands (Fig. 7): Remel 15/15; Hardy 34/35, 97%; BBL 33/35, 94%. The nine isolates that were not susceptible by BMD again fared worse due to mEs and VMEs. CA did not exceed 90% among these isolates when using Remel or Hardy MHA, and only two isolates (2/9, 22%) demonstrated CA using BBL MHA. CA also decreased below 90% when using any MHA brand among CRE (Remel 15/18, 83%; Hardy 15/18, 83%; or BBL 16/18, 89%) or *P. aeruginosa* (Hardy and BBL both 13/20, 65%) isolates. All *A. baumannii* isolates demonstrated CA with no errors observed across tested MHA brands.

## DISCUSSION

Identifying a testing method that is both practical to implement and capable of producing accurate results will reinforce the clinical utility of cefiderocol for the effective treatment of MDR GN bacterial infections. However, among our collection of MDR clinical isolates and Antibiotic Resistance Isolate bank samples, the use of commercial Remel, Hardy, and BBL MHA for DD frequently resulted in AST interpretation discrepancies when compared to use for BMD. Isolates that were not susceptible to cefiderocol via BMD commonly tested susceptible via DD across MHA brands and interpretation standards, posing a risk of under-calling resistance when using DD for organisms with MICs approaching or exceeding clinical breakpoints via BMD. While our agreement analyses were poorest when only including isolates that were classified as not susceptible to cefiderocol via BMD, analyses that included all tested isolates with available clinical breakpoints still failed to consistently achieve a CA of ≥90% between DD and reference BMD results across MHA brands and breakpoint interpretations. A potential exception to this trend involved CLSI interpretations applied to DD using BBL MHA, but BBL MHA performance concerns persisted with isolates that were not susceptible to cefiderocol by BMD. Additionally, colonies within zones of inhibition upon DD testing were most frequently observed when using BBL MHA.

The nine isolates in our collection that did not test susceptible to cefiderocol via BMD when applying CLSI, EUCAST, or FDA interpretations represented a small sub-group that poses potential concerns for microbiological interpretations and clinical practice beyond our study. We observed VME in which the DD method failed to identify resistance across brands and breakpoints, although this was least impactful with BBL MHA plates coupled with CLSI (2.2%) and EUCAST (2.6%) breakpoints and Hardy MHA with CLSI breakpoints (2.2%). These findings align with those of previous analyses (3, 21–23). Morris *et al*. proposed that DD may be a convenient alternative to BMD for cefiderocol AST but observed inconsistencies in susceptibility interpretations across CLSI, EUCAST, and FDA breakpoints for GN bacteria (21). Bonnin *et al.* raised further concern regarding DD reliability in a large study of CRE isolates plated on Biorad (Marnes la Coquette, France) MHA (3). A CA rate of 81.7% and a VME rate of 23.3% prompted a recommendation for confirmatory BMD testing in many isolates to avoid under-calling resistance (3). Additionally, Liu *et al.* evaluated the correlation between cefiderocol AST DD and BMD results for a collection of 468 *A*. *baumannii* complex isolates and found that DD was unable to accurately classify all six isolates with cefiderocol MICs ≥ 8 µg/mL as resistant (22). Jeannot *et al.* published similar findings with 97 *Acinetobacter* spp. strains and Mast Diagnostic (Merseyside, United Kingdom), Liofilchem (Roseto degli Abruzzi, Italy), and Oxoid (Thermo Fisher Scientific, Basingstoke, United Kingdom) cefiderocol 30 µg disks plus Becton Dickinson MHA and suggested that while DD may be a helpful tool for AST screening for cefiderocol-susceptible strains, interpretations of strains with zones of inhibition ≤22 mm via DD should be confirmed with BMD (23). Others have echoed this suggestion for BMD confirmatory testing when DD results fall within areas of technical uncertainty for a variety of GN bacteria (4, 24, 25). The CLSI M100 also includes a related cautionary comment outlining the potential for inaccurate testing and interpretation (4). Combined with our analyses, these findings highlight concerns with the use of DD in the pursuit of accurately identifying cefiderocol resistance.

In addition to discrepancies between DD and BMD, variability by MHA brand alone could impede identification of accurate and reproducible results between laboratories. For the species included in our study, this was most worrisome among *P. aeruginosa* and CRE isolates; 25% and almost 20% of these isolates demonstrated at least one DD breakpoint interpretation dependent on the MHA brand, respectively. Inter-brand variation in zones of diffusion >10 mm was also most common among isolates from these sub-groups, especially when comparing BBL MHA to Remel or Hardy MHA. Our collection of Enterobacterales isolates was enriched for carbapenem resistance. However, we observed errors when using all three commercial MHAs for disk diffusion that compromised an achievement of ≥90% CA regardless of BMD AST interpretation. Altogether, these observations raise concern for the likelihood of VME and failure to reliably identify cefiderocol resistance when using DD for organisms that are not found to be susceptible to cefiderocol via BMD. No differences in interpretation were noted for *S. maltophilia* or *A. baumannii* complex isolates, but these were all cefiderocol-susceptible via BMD, which again raises suspicion that DD cannot reliably differentiate resistant isolates across media. Similarly, while clinical breakpoints do not exist for *B. cepacia* complex, we observed very low modal MICs and large mean zone diameters that were similar across MHA brands. Potter *et al*. previously investigated the impact of the MHA brand on DD interpretation and found similar discrepancies in zones of inhibition between Hardy (Santa Monica, CA) and BD (Franklin Lakes, NJ) MHA, which resulted in interpretation differences for 8.7% (2/23) of their *P. aeruginosa* isolates using CLSI breakpoints (9).

Inconsistent AST results between MHA brands may be a consequence of the interplay between a lack of iron standardization in MHA plates and the unique cell entry method of cefiderocol as a siderophore cephalosporin (2). In addition to discrepancies with DD, issues with cefiderocol BMD using both commercial and reference-made plates (e.g., trailing endpoints) have been reported (2, 7, 26). Members of CLSI recently published a letter raising awareness of inconsistencies in BMD testing, emphasizing that media variability in iron concentration and inoculum may contribute to high error rates (2). We did not further explore this issue as the focus of this study was to highlight practical challenges with media used for DD, but it is likely that similar discrepancies would be seen in these isolates with BMD testing compared across commercial media from multiple manufacturers. The absence of cefiderocol resistance observed among our CP-CRE isolates may be explained by the likelihood that its development depends on the combined influence of multiple mechanisms beyond the expression of carbapenemases, including, but not limited to, alterations to siderophore receptors, penicillin-binding-protein-3 target modifications, and loss-of-function mutations in the *cirA* iron transporter gene (12). The utilization of iron channels offers a novel strategy for overcoming antimicrobial resistance in GN bacterial infections with limited treatment options (1). However, it also creates *in vitro* challenges to AST under conditions that do not closely mimic the low iron environment of infected tissues. Variability in BMD results related to iron content prompted warnings from both CLSI (2) and EUCAST (27) and led to the subsequent development of methods for depleting iron from broth for testing (4–6). Similar methods do not exist for commercial DD MHA, which was thought to resemble an iron-depleted environment due to the bound nature of its iron content (7, 8). This may not be accurate, and it may be important to develop quality controls or standard media to account for the iron content in commercial MHA.

Beyond reproducibility concerns, inconsistencies in cefiderocol DD results across MHA brands may impact the ability to estimate the *in vivo* clinical efficacy from AST results, ultimately impacting clinical decision-making and outcomes. While surveillance studies have shown promising *in vitro* susceptibility data for cefiderocol against a variety of GN pathogens (28), clinical data have highlighted concerning trends in clinical response, mortality rates, and the emergence of resistance (12–16). For example, Hoellinger *et al.* described a clinical response rate of 20% and a 30-day mortality rate of 60% for infection-related causes in a small study of mostly immunocompromised patients who received cefiderocol for at least 48 hours for the treatment of a variety of infections attributed to MDR non-fermenting, GN bacilli (13). Unreliable cefiderocol AST methods further complicate these trends, and impractical methods can contribute to delays in therapy for those with limited options for effective antimicrobials on account of multidrug resistance.

Our study was not without limitations. Although our collection of organisms was small and enriched for resistant isolates, there were notable errors among isolates that were not susceptible to cefiderocol via BMD. The trend of discrepancies being highest in the setting of cefiderocol resistance was observed among *P. aeruginosa* isolates and non-CP-CRE isolates in our study, but no other isolates were adjudicated as being intermediate or resistant among other tested species. Although this is true, cefiderocol resistance remains relatively rare, and thus we feel our data set still has value in highlighting a potential issue of variability by the media brand. Of note, we did observe trailing endpoints upon BMD testing in 14 isolates and inner colonies upon DD testing in eight isolates, but they only occurred simultaneously when using BBL MHA for DD. Only the observation of trailing endpoints occurred simultaneously with inter-brand discrepancies in DD interpretation, but this was demonstrated in a minority of isolates. Although CLSI (4, 29) and EUCAST (30) have described strategies for interpreting DD and BMD results despite these phenomena, opportunities remain for intra- and inter-reader variability. We sought to minimize the impact of trailing endpoints and inner colonies by using reading guidelines developed by CLSI and EUCAST (4, 7, 19, 29, 30). Additionally, while AST for each isolate was repeated three times, we used a single inoculum per isolate to minimize factors contributing to variability in our results. We were also limited in our ability to evaluate non-Enterobacterales DD results across all brands given the failure of QC with *P. aeruginosa* ATCC 27853 for Remel MHA. We feel this QC failure emphasizes the difficulty of achieving accurate cefiderocol AST results via DD across MHA since consistent results were not achieved when using a standardized organism. Despite these limitations, our results demonstrated key inconsistencies in susceptibility interpretations and CA across three MHA brands and breakpoint criteria that warrant further study to identify an accurate yet practical strategy for conducting AST for cefiderocol.

### Conclusions

There is a significant clinical need for standardizing cefiderocol susceptibility testing such that methods are accurate and reproducible. In our study, DD AST for cefiderocol frequently demonstrated interpretation discrepancies across clinical breakpoint standards and commercially available MHA when compared to BMD. Errors, including those of under-calling resistance, were most common among isolates that were not susceptible to cefiderocol by BMD, *P. aeruginosa* isolates, and non-CP-CRE isolates. Although our collection of isolates was small and enriched for resistant organisms, our findings raise broader concerns for the overall performance of DD beyond this study, especially among organisms that do not test susceptible to cefiderocol via BMD. Our results warrant caution with susceptibility interpretation via DD with commercially available MHA when cefiderocol is indicated. Future studies are needed to identify accurate yet practical methods for performing cefiderocol AST and to determine which unique medium-related DD results most accurately reflect *in vivo* efficacy.

## SUPPLEMENTAL MATERIAL

Supplemental tables jcm.01648-24-s0001.docx MHA distribution of cefiderocol BMD modal MICs and DD zones of inhibition.

ASM does not own the copyrights to Supplemental Material that may be linked to, or accessed through, an article. The authors have granted ASM a non-exclusive, world-wide license to publish the Supplemental Material files. Please contact the corresponding author directly for reuse.
