# Supplementary material for: Impact of media brand on cefiderocol disk diffusion results
Source: J Clin Microbiol. 2025 Mar 25;63(5):e01648-24. doi: 10.1128/jcm.01648-24 (PMC12077100; doi:10.1128/jcm.01648-24)
Supplement: Supplemental tables — MHA distribution of cefiderocol BMD modal MICs and DD zones of inhibition. [file jcm.01648-24-s0001.docx]

**Supplemental Tables:**

| **TABLE S1. Remel MHA Distribution of Cefiderocol BMD Modal MICs and DD Zones of Inhibition for CRE** | | | | | | | | | | | | | | | | | | | | | | | | | |
| --- | --- | --- | --- | --- | --- | --- | --- | --- | --- | --- | --- | --- | --- | --- | --- | --- | --- | --- | --- | --- | --- | --- | --- | --- | --- |
| BMD Modal MIC (µg/mL) | 128 |  |  |  |  |  |  |  |  |  |  |  |  |  |  |  |  |  |  |  |  |  |  |  |  |
|  | 64 |  |  | 1 | 2 |  |  |  |  |  |  |  |  |  |  |  |  |  |  |  |  |  |  |  |  |
|  | 32 |  |  |  |  |  |  |  |  |  |  |  |  |  |  |  |  |  |  |  |  |  |  |  |  |
|  | 16 |  |  |  |  |  |  |  |  |  |  |  |  |  | 2 |  | 1 |  |  |  |  |  |  |  |  |
|  | 8 |  |  |  |  |  |  |  |  |  |  |  |  |  |  |  |  | 1 | 1 | 1 |  |  |  |  |  |
|  | 4 |  |  |  |  |  |  |  |  |  |  |  |  |  |  |  | 1 | 2 |  |  |  |  |  |  |  |
|  | 2 |  |  |  |  |  |  |  |  |  |  |  |  |  | 1 | 2 |  | 1 | 1 |  | 4 |  |  |  |  |
|  | 1 |  |  |  |  |  |  |  |  |  |  |  |  |  |  | 2 | 1 | 1 | 1 | 2 | 2 |  |  | 1 | 2 |
|  | 0.5 |  |  |  |  |  |  |  |  |  |  |  |  |  |  |  |  |  | 8 | 3 |  | 1 |  |  |  |
|  | 0.25 |  |  |  |  |  |  |  |  |  |  |  |  |  |  |  |  |  |  | 3 | 2 | 1 | 2 | 1 |  |
|  | 0.125 |  |  |  |  |  |  |  |  |  |  |  |  |  |  |  |  |  |  |  |  |  |  |  |  |
|  | 0.063 |  |  |  |  |  |  |  |  |  |  |  |  |  |  |  |  |  |  |  |  |  |  |  |  |
|  |  | 7 | 8 | 9 | 10 | 11 | 12 | 13 | 14 | 15 | 16 | 17 | 18 | 19 | 20 | 21 | 22 | 23 | 24 | 25 | 26 | 27 | 28 | 29 | 30 |
| DD Zone of Inhibition (mm) | | | | | | | | | | | | | | | | | | | | | | | | | |

BMD, broth microdilution; CRE, Carbapenem-resistant Enterobacterales; DD, disk diffusion; MHA, Mueller-Hinton agar; MIC, minimum inhibitory concentration. Dashed red lines denote cefiderocol breakpoints for Enterobacterales from both the CLSI Performance Standards for Antimicrobial Susceptibility Testing, 34th ed and the FDA Identified Breakpoints for Cefiderocol Injection, last updated January 31, 2023. Solid blue lines denote cefiderocol breakpoints for Enterobacterales from the EUCAST Breakpoint tables for interpretation of MICs and zone diameters, version 14.0.

| **TABLE S2. Hardy MHA Distribution of Cefiderocol BMD Modal MICs and DD Zones of Inhibition for CRE** | | | | | | | | | | | | | | | | | | | | | | | | | |
| --- | --- | --- | --- | --- | --- | --- | --- | --- | --- | --- | --- | --- | --- | --- | --- | --- | --- | --- | --- | --- | --- | --- | --- | --- | --- |
| BMD Modal MIC (µg/mL) | 128 |  |  |  |  |  |  |  |  |  |  |  |  |  |  |  |  |  |  |  |  |  |  |  |  |
|  | 64 |  |  | 1 | 2 |  |  |  |  |  |  |  |  |  |  |  |  |  |  |  |  |  |  |  |  |
|  | 32 |  |  |  |  |  |  |  |  |  |  |  |  |  |  |  |  |  |  |  |  |  |  |  |  |
|  | 16 |  |  |  |  |  |  |  |  |  |  |  |  |  | 2 | 1 |  |  |  |  |  |  |  |  |  |
|  | 8 |  |  |  |  |  |  |  |  |  |  |  |  |  | 2 |  | 1 |  |  |  |  |  |  |  |  |
|  | 4 |  |  |  |  |  |  |  |  |  |  |  |  |  | 1 | 1 |  |  | 1 |  |  |  |  |  |  |
|  | 2 |  |  |  |  |  |  |  |  |  |  |  |  | 1 | 2 |  |  | 4 |  | 2 |  |  |  |  |  |
|  | 1 |  |  |  |  |  |  |  |  |  |  |  |  |  | 1 | 2 |  | 2 |  | 3 |  | 1 | 3 |  |  |
|  | 0.5 |  |  |  |  |  |  |  |  |  |  |  |  |  |  |  |  | 1 | 3 | 6 | 2 |  |  |  |  |
|  | 0.25 |  |  |  |  |  |  |  |  |  |  |  |  |  |  |  |  |  | 2 |  | 3 | 1 | 2 | 1 |  |
|  | 0.125 |  |  |  |  |  |  |  |  |  |  |  |  |  |  |  |  |  |  |  |  |  |  |  |  |
|  | 0.063 |  |  |  |  |  |  |  |  |  |  |  |  |  |  |  |  |  |  |  |  |  |  |  |  |
|  |  | 7 | 8 | 9 | 10 | 11 | 12 | 13 | 14 | 15 | 16 | 17 | 18 | 19 | 20 | 21 | 22 | 23 | 24 | 25 | 26 | 27 | 28 | 29 | 30 |
| DD Zone of Inhibition (mm) | | | | | | | | | | | | | | | | | | | | | | | | | |

BMD, broth microdilution; CRE, Carbapenem-resistant Enterobacterales; DD, disk diffusion; MHA, Mueller-Hinton agar; MIC, minimum inhibitory concentration. Dashed red lines denote cefiderocol breakpoints for Enterobacterales from both the CLSI Performance Standards for Antimicrobial Susceptibility Testing, 34th ed and the FDA Identified Breakpoints for Cefiderocol Injection, last updated January 31, 2023. Solid blue lines denote cefiderocol breakpoints for Enterobacterales from the EUCAST Breakpoint tables for interpretation of MICs and zone diameters, version 14.0.

| **TABLE S3. BBL MHA Distribution of Cefiderocol BMD Modal MICs and DD Zones of Inhibition for CRE** | | | | | | | | | | | | | | | | | | | | | | | | | |
| --- | --- | --- | --- | --- | --- | --- | --- | --- | --- | --- | --- | --- | --- | --- | --- | --- | --- | --- | --- | --- | --- | --- | --- | --- | --- |
| BMD Modal MIC (µg/mL) | 128 |  |  |  |  |  |  |  |  |  |  |  |  |  |  |  |  |  |  |  |  |  |  |  |  |
|  | 64 |  |  | 1 | 2 |  |  |  |  |  |  |  |  |  |  |  |  |  |  |  |  |  |  |  |  |
|  | 32 |  |  |  |  |  |  |  |  |  |  |  |  |  |  |  |  |  |  |  |  |  |  |  |  |
|  | 16 |  |  |  |  |  |  |  |  |  |  |  |  | 2 |  | 1 |  |  |  |  |  |  |  |  |  |
|  | 8 |  |  |  | 3 |  |  |  |  |  |  |  |  |  |  |  |  |  |  |  |  |  |  |  |  |
|  | 4 |  |  |  |  |  |  |  |  |  |  |  | 2 |  | 1 |  |  |  |  |  |  |  |  |  |  |
|  | 2 |  |  |  |  |  |  |  |  |  |  |  | 2 |  |  | 3 | 2 | 1 | 1 |  |  |  |  |  |  |
|  | 1 |  |  |  |  |  |  |  |  |  |  |  |  |  | 1 | 2 |  | 5 |  | 1 | 3 |  |  |  |  |
|  | 0.5 |  |  |  |  |  |  |  |  |  |  |  |  |  |  |  |  | 5 | 3 | 3 |  | 1 |  |  |  |
|  | 0.25 |  |  |  |  |  |  |  |  |  |  |  |  |  |  |  |  |  | 3 | 2 | 1 |  | 2 | 1 |  |
|  | 0.125 |  |  |  |  |  |  |  |  |  |  |  |  |  |  |  |  |  |  |  |  |  |  |  |  |
|  | 0.063 |  |  |  |  |  |  |  |  |  |  |  |  |  |  |  |  |  |  |  |  |  |  |  |  |
|  |  | 7 | 8 | 9 | 10 | 11 | 12 | 13 | 14 | 15 | 16 | 17 | 18 | 19 | 20 | 21 | 22 | 23 | 24 | 25 | 26 | 27 | 28 | 29 | 30 |
| DD Zone of Inhibition (mm) | | | | | | | | | | | | | | | | | | | | | | | | | |

BMD, broth microdilution; CRE, Carbapenem-resistant Enterobacterales; DD, disk diffusion; MHA, Mueller-Hinton agar; MIC, minimum inhibitory concentration. Dashed red lines denote cefiderocol breakpoints for Enterobacterales from both the CLSI Performance Standards for Antimicrobial Susceptibility Testing, 34th ed and the FDA Identified Breakpoints for Cefiderocol Injection, last updated January 31, 2023. Solid blue lines denote cefiderocol breakpoints for Enterobacterales from the EUCAST Breakpoint tables for interpretation of MICs and zone diameters, version 14.0.

| **TABLE S4. Hardy MHA Distribution of Cefiderocol BMD Modal MICs and DD Zones of Inhibition for PsA** | | | | | | | | | | | | | | | | | | | | | | | | | | | | | |
| --- | --- | --- | --- | --- | --- | --- | --- | --- | --- | --- | --- | --- | --- | --- | --- | --- | --- | --- | --- | --- | --- | --- | --- | --- | --- | --- | --- | --- | --- |
| BMD Modal MIC (µg/mL) | 128 |  |  |  |  |  |  |  |  |  |  |  |  |  |  |  |  |  |  |  |  |  |  |  |  |  |  |  |  |
|  | 64 |  |  |  |  |  |  |  |  |  |  |  |  |  |  |  |  |  |  |  |  |  |  |  |  |  |  |  |  |
|  | 32 |  |  |  |  |  |  |  |  |  |  |  |  |  |  |  |  |  |  |  |  |  |  |  |  |  |  |  |  |
|  | 16 |  |  |  |  |  |  |  |  |  |  |  |  |  |  |  |  |  |  |  |  |  |  |  |  |  |  |  |  |
|  | 8 |  |  |  |  |  |  |  | 2 | 2 |  | 1 |  |  |  |  | 1 |  |  |  |  |  |  |  |  |  |  |  |  |
|  | 4 |  |  |  |  |  |  |  | 2 |  |  | 1 |  |  |  | 1 | 1 |  |  |  |  |  |  | 1 |  |  |  |  |  |
|  | 2 |  |  |  |  |  |  |  |  |  |  |  |  | 1 | 1 | 1 |  |  |  |  |  | 2 | 1 |  |  |  |  |  |  |
|  | 1 |  |  |  |  |  |  |  |  |  | 1 | 1 |  |  |  |  | 2 | 2 |  |  |  |  |  |  |  |  |  |  |  |
|  | 0.5 |  |  |  |  |  |  |  |  |  |  |  |  | 1 |  |  |  | 1 | 1 | 2 | 1 | 2 |  | 1 |  |  |  |  |  |
|  | 0.25 |  |  |  |  |  |  |  |  | 1 | 2 |  | 2 |  |  | 6 | 2 |  |  | 5 | 2 | 1 |  |  |  |  |  |  |  |
|  | 0.125 |  |  |  |  |  |  |  |  |  |  |  |  | 1 | 1 |  |  |  | 1 |  |  |  |  |  |  |  |  |  |  |
|  | 0.063 |  |  |  |  |  |  |  |  |  |  |  |  |  |  |  |  |  |  |  |  |  |  |  |  |  |  |  |  |
|  | <0.063 |  |  |  |  |  |  |  |  |  |  |  |  |  |  |  |  |  |  |  |  |  |  |  |  |  | 2 | 1 |  |
|  |  | 12 | 13 | 14 | 15 | 16 | 17 | 18 | 19 | 20 | 21 | 22 | 23 | 24 | 25 | 26 | 27 | 28 | 29 | 30 | 31 | 32 | 33 | 34 | 35 | 36 | 37 | 38 | 39 |
| DD Zone of Inhibition (mm) | | | | | | | | | | | | | | | | | | | | | | | | | | | | | |

BMD, broth microdilution; DD, disk diffusion; MHA, Mueller-Hinton agar; MIC, minimum inhibitory concentration; PsA, *Pseudomonas aeruginosa.* Solid red lines denote cefiderocol breakpoints for *P. aeruginosa* from the CLSI Performance Standards for Antimicrobial Susceptibility Testing, 34th ed. The dashed red line denotes cefiderocol breakpoints for *P. aeruginosa* from both the CLSI Performance Standards for Antimicrobial Susceptibility Testing, 34th ed and the FDA Identified Breakpoints for Cefiderocol Injection, last updated January 31, 2023. Dashed blue lines represent cefiderocol breakpoints for *P. aeruginosa* from both the EUCAST Breakpoint tables for interpretation of MICs and zone diameters, version 14.0 and the FDA Identified Breakpoints for Cefiderocol Injection, last updated January 31, 2023. The solid green line denotes cefiderocol breakpoints for *P. aeruginosa* from the FDA Identified Breakpoints for Cefiderocol Injection, last updated January 31, 2023.

| **TABLE S5. BBL MHA Distribution of Cefiderocol BMD Modal MICs and DD Zones of Inhibition for PsA** | | | | | | | | | | | | | | | | | | | | | | | | | | | | | |
| --- | --- | --- | --- | --- | --- | --- | --- | --- | --- | --- | --- | --- | --- | --- | --- | --- | --- | --- | --- | --- | --- | --- | --- | --- | --- | --- | --- | --- | --- |
| BMD Modal MIC (µg/mL) | 128 |  |  |  |  |  |  |  |  |  |  |  |  |  |  |  |  |  |  |  |  |  |  |  |  |  |  |  |  |
|  | 64 |  |  |  |  |  |  |  |  |  |  |  |  |  |  |  |  |  |  |  |  |  |  |  |  |  |  |  |  |
|  | 32 |  |  |  |  |  |  |  |  |  |  |  |  |  |  |  |  |  |  |  |  |  |  |  |  |  |  |  |  |
|  | 16 |  |  |  |  |  |  |  |  |  |  |  |  |  |  |  |  |  |  |  |  |  |  |  |  |  |  |  |  |
|  | 8 |  |  | 1 | 1 | 1 | 2 | 1 |  |  |  |  |  |  |  |  |  |  |  |  |  |  |  |  |  |  |  |  |  |
|  | 4 |  |  |  |  |  | 2 | 1 |  |  |  |  |  | 1 | 1 |  |  |  | 1 |  |  |  |  |  |  |  |  |  |  |
|  | 2 |  |  | 1 | 1 |  | 1 |  |  |  |  |  |  |  |  |  | 3 |  |  |  |  |  |  |  |  |  |  |  |  |
|  | 1 |  |  |  |  |  |  | 1 |  |  |  | 1 | 2 | 2 |  |  |  |  |  |  |  |  |  |  |  |  |  |  |  |
|  | 0.5 |  |  |  |  |  |  |  |  | 1 | 1 |  |  | 1 | 3 |  |  |  |  |  |  |  | 2 |  | 1 |  |  |  |  |
|  | 0.25 |  |  |  |  |  |  |  |  |  | 4 | 3 |  | 1 | 3 | 7 | 2 |  | 1 |  |  |  |  |  |  |  |  |  |  |
|  | 0.125 |  |  |  |  |  |  |  |  |  |  |  |  |  | 2 |  |  | 1 |  |  |  |  |  |  |  |  |  |  |  |
|  | 0.063 |  |  |  |  |  |  |  |  |  |  |  |  |  |  |  |  |  |  |  |  |  |  |  |  |  |  |  |  |
|  | <0.063 |  |  |  |  |  |  |  |  |  |  |  |  |  |  |  |  |  |  |  |  |  |  |  |  |  |  | 2 | 1 |
|  |  | 12 | 13 | 14 | 15 | 16 | 17 | 18 | 19 | 20 | 21 | 22 | 23 | 24 | 25 | 26 | 27 | 28 | 29 | 30 | 31 | 32 | 33 | 34 | 35 | 36 | 37 | 38 | 39 |
| DD Zone of Inhibition (mm) | | | | | | | | | | | | | | | | | | | | | | | | | | | | | |

BMD, broth microdilution; DD, disk diffusion; MHA, Mueller-Hinton agar; MIC, minimum inhibitory concentration; PsA, *Pseudomonas aeruginosa.* Solid red lines denote cefiderocol breakpoints for *P. aeruginosa* from the CLSI Performance Standards for Antimicrobial Susceptibility Testing, 34th ed. The dashed red line denotes cefiderocol breakpoints for *P. aeruginosa* from both the CLSI Performance Standards for Antimicrobial Susceptibility Testing, 34th ed and the FDA Identified Breakpoints for Cefiderocol Injection, last updated January 31, 2023. Dashed blue lines represent cefiderocol breakpoints for *P. aeruginosa* from both the EUCAST Breakpoint tables for interpretation of MICs and zone diameters, version 14.0 and the FDA Identified Breakpoints for Cefiderocol Injection, last updated January 31, 2023. The solid green line denotes cefiderocol breakpoints for *P. aeruginosa* from the FDA Identified Breakpoints for Cefiderocol Injection, last updated January 31, 2023.

| **TABLE S6. Hardy MHA Distribution of Cefiderocol BMD Modal MICs and DD Zones of Inhibition for AbC** | | | | | | | | | | | | | | | | | | | | | | |
| --- | --- | --- | --- | --- | --- | --- | --- | --- | --- | --- | --- | --- | --- | --- | --- | --- | --- | --- | --- | --- | --- | --- |
| BMD Modal MIC (µg/mL) | 128 |  |  |  |  |  |  |  |  |  |  |  |  |  |  |  |  |  |  |  |  |  |
|  | 64 |  |  |  |  |  |  |  |  |  |  |  |  |  |  |  |  |  |  |  |  |  |
|  | 32 |  |  |  |  |  |  |  |  |  |  |  |  |  |  |  |  |  |  |  |  |  |
|  | 16 |  |  |  |  |  |  |  |  |  |  |  |  |  |  |  |  |  |  |  |  |  |
|  | 8 |  |  |  |  |  |  |  |  |  |  |  |  |  |  |  |  |  |  |  |  |  |
|  | 4 |  |  |  |  |  |  |  |  |  |  |  |  |  |  |  |  |  |  |  |  |  |
|  | 2 |  |  |  |  |  |  |  |  |  |  |  |  |  |  |  |  |  |  |  |  |  |
|  | 1 |  |  |  |  |  |  |  |  |  |  |  |  |  |  |  |  |  |  |  |  |  |
|  | 0.5 |  |  |  |  |  |  |  |  |  | 5 | 1 |  | 6 |  |  |  |  |  |  |  |  |
|  | 0.25 |  |  |  |  |  |  |  |  |  |  |  |  | 2 | 1 | 2 | 1 |  |  |  |  |  |
|  | 0.125 |  |  |  |  |  |  |  |  |  |  |  |  |  |  |  |  |  |  |  |  |  |
|  | 0.063 |  |  |  |  |  |  |  |  |  |  |  |  |  |  |  |  |  |  |  |  |  |
|  |  | 10 | 11 | 12 | 13 | 14 | 15 | 16 | 17 | 18 | 19 | 20 | 21 | 22 | 23 | 24 | 25 | 26 | 27 | 28 | 29 | 30 |
| DD Zone of Inhibition (mm) | | | | | | | | | | | | | | | | | | | | | | |

AbC, *Acinetobacter baumannii* complex; BMD, broth microdilution*;* DD, disk diffusion; MHA, Mueller-Hinton agar; MIC, minimum inhibitory concentration. Solid red lines represent *Acinetobacter* breakpoints for cefiderocol from the CLSI Performance Standards for Antimicrobial Susceptibility Testing, 34th ed. Solid blue lines represent *Acinetobacter* breakpoints for cefiderocol from the EUCAST Breakpoint tables for interpretation of MICs and zone diameters, version 14.0. Solid green lines represent *Acinetobacter* breakpoints for cefiderocol from the FDA Identified Breakpoints for Cefiderocol Injection, last updated January 31, 2023.

| **TABLE S7. BBL MHA Distribution of Cefiderocol BMD Modal MICs and DD Zones of Inhibition for AbC** | | | | | | | | | | | | | | | | | | | | | | |
| --- | --- | --- | --- | --- | --- | --- | --- | --- | --- | --- | --- | --- | --- | --- | --- | --- | --- | --- | --- | --- | --- | --- |
| BMD Modal MIC (µg/mL) | 128 |  |  |  |  |  |  |  |  |  |  |  |  |  |  |  |  |  |  |  |  |  |
|  | 64 |  |  |  |  |  |  |  |  |  |  |  |  |  |  |  |  |  |  |  |  |  |
|  | 32 |  |  |  |  |  |  |  |  |  |  |  |  |  |  |  |  |  |  |  |  |  |
|  | 16 |  |  |  |  |  |  |  |  |  |  |  |  |  |  |  |  |  |  |  |  |  |
|  | 8 |  |  |  |  |  |  |  |  |  |  |  |  |  |  |  |  |  |  |  |  |  |
|  | 4 |  |  |  |  |  |  |  |  |  |  |  |  |  |  |  |  |  |  |  |  |  |
|  | 2 |  |  |  |  |  |  |  |  |  |  |  |  |  |  |  |  |  |  |  |  |  |
|  | 1 |  |  |  |  |  |  |  |  |  |  |  |  |  |  |  |  |  |  |  |  |  |
|  | 0.5 |  |  |  |  |  |  |  |  |  |  | 2 | 7 |  | 2 | 1 |  |  |  |  |  |  |
|  | 0.25 |  |  |  |  |  |  |  |  |  |  | 1 | 1 | 1 | 2 |  |  | 1 |  |  |  |  |
|  | 0.125 |  |  |  |  |  |  |  |  |  |  |  |  |  |  |  |  |  |  |  |  |  |
|  | 0.063 |  |  |  |  |  |  |  |  |  |  |  |  |  |  |  |  |  |  |  |  |  |
|  |  | 10 | 11 | 12 | 13 | 14 | 15 | 16 | 17 | 18 | 19 | 20 | 21 | 22 | 23 | 24 | 25 | 26 | 27 | 28 | 29 | 30 |
| DD Zone of Inhibition (mm) | | | | | | | | | | | | | | | | | | | | | | |

AbC, *Acinetobacter baumannii* complex; BMD, broth microdilution*;* DD, disk diffusion; MHA, Mueller-Hinton agar; MIC, minimum inhibitory concentration. Solid red lines represent *Acinetobacter* breakpoints for cefiderocol from the CLSI Performance Standards for Antimicrobial Susceptibility Testing, 34th ed. Solid blue lines represent *Acinetobacter* breakpoints for cefiderocol from the EUCAST Breakpoint tables for interpretation of MICs and zone diameters, version 14.0. Solid green lines represent *Acinetobacter* breakpoints for cefiderocol from the FDA Identified Breakpoints for Cefiderocol Injection, last updated January 31, 2023.

| **TABLE S8. Hardy MHA Distribution of Cefiderocol BMD Modal MICs and DD Zones of Inhibition for Sm** | | | | | | | | | | | | | | | | | | | | | |
| --- | --- | --- | --- | --- | --- | --- | --- | --- | --- | --- | --- | --- | --- | --- | --- | --- | --- | --- | --- | --- | --- |
| BMD Modal MIC (µg/mL) | 128 |  |  |  |  |  |  |  |  |  |  |  |  |  |  |  |  |  |  |  |  |
|  | 64 |  |  |  |  |  |  |  |  |  |  |  |  |  |  |  |  |  |  |  |  |
|  | 32 |  |  |  |  |  |  |  |  |  |  |  |  |  |  |  |  |  |  |  |  |
|  | 16 |  |  |  |  |  |  |  |  |  |  |  |  |  |  |  |  |  |  |  |  |
|  | 8 |  |  |  |  |  |  |  |  |  |  |  |  |  |  |  |  |  |  |  |  |
|  | 4 |  |  |  |  |  |  |  |  |  |  |  |  |  |  |  |  |  |  |  |  |
|  | 2 |  |  |  |  |  |  |  |  |  |  |  |  |  |  |  |  |  |  |  |  |
|  | 1 |  |  |  |  |  |  |  |  |  |  |  |  |  |  |  |  |  |  |  |  |
|  | 0.5 |  |  |  |  |  |  |  |  |  |  |  |  |  |  |  |  |  |  |  |  |
|  | 0.25 |  |  |  |  |  |  |  |  |  |  |  |  |  |  |  | 3 |  |  | 1 | 2 |
|  | 0.125 |  |  |  |  |  |  |  |  |  |  |  |  |  |  |  |  |  |  |  |  |
|  | 0.063 |  |  |  |  |  |  |  |  |  |  |  |  |  |  |  |  |  |  |  |  |
|  |  | 14 | 15 | 16 | 17 | 18 | 19 | 20 | 21 | 22 | 23 | 24 | 25 | 26 | 27 | 28 | 29 | 30 | 31 | 32 | 33 |
| DD Zone of Inhibition (mm) | | | | | | | | | | | | | | | | | | | | | |

BMD, broth microdilution*;* DD, disk diffusion; MHA, Mueller-Hinton agar; MIC, minimum inhibitory concentration; Sm; *Stenotrophomonas maltophilia*. Solid red lines represent *Stenotrophomonas* breakpoints for cefiderocol from the CLSI Performance Standards for Antimicrobial Susceptibility Testing, 34th ed. Solid blue lines represent *Stenotrophomonas* breakpoints for cefiderocol from the EUCAST Breakpoint tables for interpretation of MICs and zone diameters, version 14.0.

| **TABLE S9. BBL MHA Distribution of Cefiderocol BMD Modal MICs and DD Zones of Inhibition for Sm** | | | | | | | | | | | | | | | | | | | | | |
| --- | --- | --- | --- | --- | --- | --- | --- | --- | --- | --- | --- | --- | --- | --- | --- | --- | --- | --- | --- | --- | --- |
| BMD Modal MIC (µg/mL) | 128 |  |  |  |  |  |  |  |  |  |  |  |  |  |  |  |  |  |  |  |  |
|  | 64 |  |  |  |  |  |  |  |  |  |  |  |  |  |  |  |  |  |  |  |  |
|  | 32 |  |  |  |  |  |  |  |  |  |  |  |  |  |  |  |  |  |  |  |  |
|  | 16 |  |  |  |  |  |  |  |  |  |  |  |  |  |  |  |  |  |  |  |  |
|  | 8 |  |  |  |  |  |  |  |  |  |  |  |  |  |  |  |  |  |  |  |  |
|  | 4 |  |  |  |  |  |  |  |  |  |  |  |  |  |  |  |  |  |  |  |  |
|  | 2 |  |  |  |  |  |  |  |  |  |  |  |  |  |  |  |  |  |  |  |  |
|  | 1 |  |  |  |  |  |  |  |  |  |  |  |  |  |  |  |  |  |  |  |  |
|  | 0.5 |  |  |  |  |  |  |  |  |  |  |  |  |  |  |  |  |  |  |  |  |
|  | 0.25 |  |  |  |  |  |  |  |  |  |  |  |  |  |  |  |  | 3 | 3 |  |  |
|  | 0.125 |  |  |  |  |  |  |  |  |  |  |  |  |  |  |  |  |  |  |  |  |
|  | 0.063 |  |  |  |  |  |  |  |  |  |  |  |  |  |  |  |  |  |  |  |  |
|  |  | 14 | 15 | 16 | 17 | 18 | 19 | 20 | 21 | 22 | 23 | 24 | 25 | 26 | 27 | 28 | 29 | 30 | 31 | 32 | 33 |
| DD Zone of Inhibition (mm) | | | | | | | | | | | | | | | | | | | | | |

BMD, broth microdilution*;* DD, disk diffusion; MHA, Mueller-Hinton agar; MIC, minimum inhibitory concentration; Sm; *Stenotrophomonas maltophilia*. Solid red lines represent *Stenotrophomonas* breakpoints for cefiderocol from the CLSI Performance Standards for Antimicrobial Susceptibility Testing, 34th ed. Solid blue lines represent *Stenotrophomonas* breakpoints for cefiderocol from the EUCAST Breakpoint tables for interpretation of MICs and zone diameters, version 14.0.

| **TABLE S10. Hardy MHA Distribution of Cefiderocol BMD Modal MICs and DD Zones of Inhibition for BcC** | | | | | | | | | | | | | | | | | | | | | | |
| --- | --- | --- | --- | --- | --- | --- | --- | --- | --- | --- | --- | --- | --- | --- | --- | --- | --- | --- | --- | --- | --- | --- |
| BMD Modal MIC (µg/mL) | 128 |  |  |  |  |  |  |  |  |  |  |  |  |  |  |  |  |  |  |  |  |  |
|  | 64 |  |  |  |  |  |  |  |  |  |  |  |  |  |  |  |  |  |  |  |  |  |
|  | 32 |  |  |  |  |  |  |  |  |  |  |  |  |  |  |  |  |  |  |  |  |  |
|  | 16 |  |  |  |  |  |  |  |  |  |  |  |  |  |  |  |  |  |  |  |  |  |
|  | 8 |  |  |  |  |  |  |  |  |  |  |  |  |  |  |  |  |  |  |  |  |  |
|  | 4 |  |  |  |  |  |  |  |  |  |  |  |  |  |  |  |  |  |  |  |  |  |
|  | 2 |  |  |  |  |  |  |  |  |  |  |  |  |  |  |  |  |  |  |  |  |  |
|  | 1 |  |  |  |  |  |  |  |  |  |  |  |  |  |  |  |  |  |  |  |  |  |
|  | 0.5 |  |  |  |  |  |  |  |  |  |  |  |  |  |  |  |  |  |  |  |  |  |
|  | 0.25 |  |  |  |  |  |  |  |  |  |  |  |  |  |  |  |  |  |  |  |  |  |
|  | 0.125 |  |  |  |  |  |  |  |  |  |  |  |  |  |  |  |  |  |  |  |  |  |
|  | 0.063 |  |  |  |  |  |  |  |  |  |  |  |  |  |  |  |  |  |  |  |  |  |
|  | <0.063 |  |  |  |  |  |  |  |  |  |  |  |  | 2 | 1 | 2 | 1 |  |  |  |  |  |
|  |  | 22 | 23 | 24 | 25 | 26 | 27 | 28 | 29 | 30 | 31 | 32 | 33 | 34 | 35 | 36 | 37 | 38 | 39 | 40 | 41 | 42 |
| DD Zone of Inhibition (mm) | | | | | | | | | | | | | | | | | | | | | | |

BcC, *Burkholderia cepacia* complex; BMD, broth microdilution*;* DD, disk diffusion; MHA, Mueller-Hinton agar; MIC, minimum inhibitory concentration.

| **TABLE S11. BBL MHA Distribution of Cefiderocol BMD Modal MICs and DD Zones of Inhibition for BcC** | | | | | | | | | | | | | | | | | | | | | | |
| --- | --- | --- | --- | --- | --- | --- | --- | --- | --- | --- | --- | --- | --- | --- | --- | --- | --- | --- | --- | --- | --- | --- |
| BMD Modal MIC (µg/mL) | 128 |  |  |  |  |  |  |  |  |  |  |  |  |  |  |  |  |  |  |  |  |  |
|  | 64 |  |  |  |  |  |  |  |  |  |  |  |  |  |  |  |  |  |  |  |  |  |
|  | 32 |  |  |  |  |  |  |  |  |  |  |  |  |  |  |  |  |  |  |  |  |  |
|  | 16 |  |  |  |  |  |  |  |  |  |  |  |  |  |  |  |  |  |  |  |  |  |
|  | 8 |  |  |  |  |  |  |  |  |  |  |  |  |  |  |  |  |  |  |  |  |  |
|  | 4 |  |  |  |  |  |  |  |  |  |  |  |  |  |  |  |  |  |  |  |  |  |
|  | 2 |  |  |  |  |  |  |  |  |  |  |  |  |  |  |  |  |  |  |  |  |  |
|  | 1 |  |  |  |  |  |  |  |  |  |  |  |  |  |  |  |  |  |  |  |  |  |
|  | 0.5 |  |  |  |  |  |  |  |  |  |  |  |  |  |  |  |  |  |  |  |  |  |
|  | 0.25 |  |  |  |  |  |  |  |  |  |  |  |  |  |  |  |  |  |  |  |  |  |
|  | 0.125 |  |  |  |  |  |  |  |  |  |  |  |  |  |  |  |  |  |  |  |  |  |
|  | 0.063 |  |  |  |  |  |  |  |  |  |  |  |  |  |  |  |  |  |  |  |  |  |
|  | <0.063 |  |  |  |  |  |  |  |  |  |  |  |  |  |  | 1 | 3 |  |  | 1 | 1 |  |
|  |  | 22 | 23 | 24 | 25 | 26 | 27 | 28 | 29 | 30 | 31 | 32 | 33 | 34 | 35 | 36 | 37 | 38 | 39 | 40 | 41 | 42 |
| DD Zone of Inhibition (mm) | | | | | | | | | | | | | | | | | | | | | | |

BcC, *Burkholderia cepacia* complex; BMD, broth microdilution*;* DD, disk diffusion; MHA, Mueller-Hinton agar; MIC, minimum inhibitory concentration.
